# Telephone interviews among a cohort of gambling patients at the time of COVID-19

**DOI:** 10.1192/j.eurpsy.2021.2164

**Published:** 2021-08-13

**Authors:** E. Levari, A. Negri, P. Simonato, G. Tomasi, G. Branz, A. Coppola, P. Gianfranceschi, E. Leoni, P. Mistretta, M. Stefani, M. Vanzetta, O. Corazza, A. Franceschini

**Affiliations:** 1 Addiction Unit, National Health Service, Trento, Italy; 2 B. school Of Life And Medical Sciences, University of Hertfordshire, Hatfield, United Kingdom; 3 Parco Dei Tigli, Casa di Cura “Parco dei Tigli”, Teolo, Italy; 4 Ama, Associazione Auto Mutuo Aiuto, Trento, Italy; 5 Department Of Clinical And Pharmaceutical Sciences, University of Hertfordshire, Hatfield, United Kingdom

**Keywords:** Gambling, lockdown, Survey, COVID-19

## Abstract

**Introduction:**

Background: Little is known about the modifications in gambling patterns during the Covid-19 pandemic, which has shown signs of increase, particularly for individuals with preexisting gambling problems.

**Objectives:**

Our aim was to assess the behaviour of a cohort of patients in the Trentino Region.

**Methods:**

A semi structured questionnaire containing Hamilton Depression Rating Scale as well as open-ended questions on gambling activities, specifically online gambling, was administred over the telephone. The survey was administred for two months over the lockdown period (april-june 2020) and took approximately 20 minutes to complete.

**Results:**

About 50 responsens were collected. Data are currently been analyzed and will be avaiable at the time of the Congress.

**Conclusions:**

Will be show at the time of the Congress.

**Disclosure:**

No significant relationships.

